# Knowledge and Practices of Hearing Screening and Hearing Loss Management among Ear, Nose, and Throat Physicians in Saudi Arabia

**DOI:** 10.1055/s-0045-1811516

**Published:** 2026-04-01

**Authors:** Fahad Wadi S. Alanazi, Lujain Bandar Alotaibi, Yousef Aljathlany, Saleh Alabood

**Affiliations:** 1 College of Medicine, Northern Border University, Arar, Northern Borders Province, Saudi Arabia; 2 Deanship of Scientific Research, College of Medicine, Imam Mohammed Ibn Saud Islamic University, Riyadh, Riyadh Province, Saudi Arabia; 3 Department of Otolaryngology, College of Medicine, King Saud University, Riyadh, Riyadh Province, Saudi Arabia; 4 Otolaryngology–Head & Neck Surgery Unit, Department of Surgery, Security Forces Hospital, Riyadh, Riyadh Province, Saudi Arabia

**Keywords:** knowledge, practice, otolaryngology, hearing, screening, management

## Abstract

**Introduction:**

One of the main factors that lead to increased hearing loss consequences and progression is inadequate knowledge and practice of hearing screening and hearing loss management modalities. In Saudi Arabia, ear, nose, and throat (ENT) physicians are the primary source of knowledge about hearing loss and its management. The aim of the present study is to assess the knowledge and practice of hearing screening and hearing loss management among ENT physicians in Saudi Arabia.

**Objective:**

The aim of the present study is to assess the knowledge and practice of hearing screening and hearing loss management among ENT physicians in Saudi Arabia.

**Methods:**

We conducted a cross-sectional descriptive study with 106 ENT physicians working at different hospitals in Saudi Arabia from January to May 2023. A questionnaire consisting of 2 sections with 20 questions was used to assess the knowledge and practice of hearing screening and hearing loss management for children.

**Results:**

The current study showed inadequate knowledge of hearing loss and its management. The mean knowledge score was of 12.4 ± 2.12. The knowledge scores of the different types of ENT professionals were compared, and no statistically significant differences were observed ( *p* = 0.489).

**Conclusion:**

More research should be conducted to assess the practice of hearing screening in Saudi Arabia. Our recommendation is the provision of more educational sessions about the recent guidelines on hearing loss screening.

## Introduction

Hearing loss is a condition frequently encountered that progresses with age, both in terms of incidence and severity. 1 Hearing loss occurs in 1 to 3 newborns per 1 thousand births, 2 with 1 to 2 per 1 thousand suffering from permanent childhood hearing impairment. 3

According to the World Health Organization (WHO), 4 34 million children are estimated to require rehabilitation to address their hearing loss. Nearly 60% of hearing loss in children is due to preventable causes that can be avoided by implementing public health measures such as identifying and managing common ear conditions that can lead to hearing loss. 4

Early identification of hearing loss and ear diseases is critical for effective management. This requires systematic screening for the detection of hearing loss and related ear diseases in those who are at higher risk, including newborns, infants, preschoolers and schoolchildren. 5 Once hearing loss is identified, it is essential to address it as early as possible and in an appropriate manner.

The diagnosis and management of hearing loss require an interprofessional team that involves an otolaryngologist, a speech therapist, an audiologist, and a social worker. Developing a normal language and psychosocial functioning requires early detection and prompt management of hearing loss. Universal newborn hearing screening, followed by definitive audiological diagnosis and early fitting of hearing aids or cochlear implantation, has fundamentally improved prelingual hearing rehabilitation and enabled (near) normal vocal speech acquisition. 6

Despite the importance of implementing screening programs, in developing countries, including Saudi Arabia, the implementation rates are not ideal. 7 The Saudi pediatric population has a high rate of permanent hearing loss, which has been shown to be of 7.7%. 8 Between September 1997 and May 2000, an extensive random-sample study invo0lving 9,540 children under the age of 15 years was conducted in Saudi Arabia. 9 The primary goal of the study was to evaluate children regarding hearing impairment and to investigate the prevalence of sensorineural hearing loss (SNHL). According to the findings, 13% of the examined children presented hearing impairment. 9

A previous study 10 also conducted in Saudi Arabia has demonstrated that newborns with severe to profound hearing loss who undergo hearing screening at birth are significantly more likely to be diagnosed and treated in a timely manner.

One of the main factors that lead to increased hearing loss consequences and progression is inadequate knowledge and practice of hearing screening and hearing loss management modalities. The literature on the assessment of these aspects is limited, especially regarding studies conducted by ear, nose, and throat (ENT) physicians in Saudi Arabia. The current study is the first to assess the knowledge and practice of hearing screening and hearing loss management among ENT physicians in Saudi Arabia.

## Methods

### Study Design

After receiving approval from the institutional Review Board, the current study was conducted in Saudi Arabia with a cross-sectional study design through an electronic survey between January and May 2023. The electronic survey was distributed through social media to multiple groups of ENT physicians. The questionnaire was adapted from the study by Zaitoun et al., 11 and was used to investigate the level of knowledge and the practice of hearing loss management among ENT physicians in Saudi Arabia. The questionnaire contained 20 questions divided into 2 sections: the first section included 7 questions about the respondents' demographics, such as specialty, years of experience, age, gender, and practice setting, and the second section included 13 questions about the level of knowledge and practice regarding hearing loss and its management. All of the questions were written in English and ranged from yes/no to multiple choice questions, as well as those that required short written answers.

### Statistical Analysis

The data collected were cleaned and then analyzed using the IBM SPSS Statistics for Windows (IBM Corp.) software, version 23.0. Data analysis was performed by an independent biostatistician. The categorical variables were expressed as frequencies and percentages, and the continuous variables, as mean and standard deviation values. The Pearson's Chi-squared test was used to evaluate statistical relationships involving the categorical variables. Values of *p* ≤ 0.05 were considered statistically significant.

## Results

The survey was filled out by 106 practicing ENT physicians in various provinces of Saudi Arabia: 35 (33%) ENT consultants, 27 (25.5%) ENT specialists, and 43 (40.6%) ENT residents. Most of the respondents (76; 71.7%) were of the male gender, and 42 (39.6%) had 2 to 5 years of experience. Regarding age, 42 (39.6%) were aged between 23 and 29 years, and 38 (35.8%), between 30 and 39 years. Nearly half of the participants (50; 47.2%) worked in the Saudi Ministry of Health, 27 (25.5%), in military medical services, and 19 (17.9%), in medical schools or universities. As for the region of practice within Saudi Arabia, 44 (41.5%) were from the Central region, 30 (28.3%), from the Western region, and 18 (17%), from the Eastern region ( Table 1 ).

**Table 1:** Sociodemographic data of the study sample

		N	%
Type of ear, nose, and throat (ENT) professional	Consultant	35	33.0
	Specialist	27	25.5
	Resident	43	40.6
	Teaching assistant	1	0.9
Gender	Female	30	28.3
	Male	76	71.7
Years of experience	< 1	15	14.2
	2–5	42	39.6
	6–10	24	22.6
	> 11	25	23.6
Age group (years)	26–29	42	39.6
	30–39	38	35.8
	40–49	16	15.1
	50–59	6	5.7
	> 60	4	3.8
Workplace	Medical school/university	19	17.9
	Military medical services	27	25.5
	Ministry of Health	50	47.2
	Private practice	4	3.8
	Other	6	5.7
Region of practice in Saudi Arabia	Central	44	41.5
	Eastern	18	17.0
	Northern	3	2.8
	Southern	11	10.4
	Western	30	28.3

The practices and experiences of the respondents related to hearing screening are shown in Table 2 . When we asked the participants to provide an estimate of the number of newborns who had received hearing screening results indicative of impairment during the last year, 44 (41.5%) reported that they could not do it, 18 (17%) reported more than 10, 14 (13.2%), 11 to 50, and 16 (15.1%), more than 50. As for the estimated number of children with permanent sensorineural hearing loss (SNHL) who consulted with the respondents in the previous year, 34 (32.1%) reported “none”, 42 (39.6%), 1 to 10, 23 (21.7%), 11 to 50, and 7 (6.6%), more than 50. When asked where they would routinely refer to the family of a child with confirmed permanent hearing loss, 36 (34%) reported a specialized center for audiology and otology, 7 (6.6%), any tertiary hospital, whereas 3 (2.8%) reported that they would hold the consultation themselves. A total of 35 (33%) respondents reported that they faced difficulty finding places that provide reliable audiology-related services. Regarding Saudi Arabia's Early Hearing Detection and Intervention Program, 86 (81.1%) of the respondents were aware of its existence, and 102 (96.2%) agreed that it is very important to screen all newborns for permanent hearing loss. More than half of the participants (54; 50.9%) did not agree that hearing screening causes parents to feel excessive anxiety and/or concern, and only 33 (31.1%) were aware that different health insurance plans cover audiological services, including hearing aids and cochlear implantation.

**Table 2:** Practices, awareness, and experiences related to hearing screening and programs

		N	%
Estimate the number of newborns who received hearing screening results indicative of impairment during the last year.	< 10	18	17.0
	11–50	14	13.2
	> 50	16	15.1
	Too many to estimate	7	6.6
	I do not know	44	41.5
	Only when I receive referrals	7	6.6
Number of children with permanent sensorineural hearing loss (SNHL) you have examined in your practice last year.	None	34	32.1
	1–10	42	39.6
	11–50	23	21.7
	> 50	7	6.6
To where do routinely refer the family of a child with a confirmed permanent hearing loss?	An audiologist in the same hospital	34	32.1
	An otologist in the same hospital	35	33.0
	A tertiary hospital	7	6.6
	A specialized center for audiology and otology	36	34.0
	I hold the consultation myself	3	2.8
	Others	3	2.8
	I do not know	4	3.7
Do you face difficulty finding places that provide reliable audiology related services?	No	71	67.0
	Yes	35	33.0
Is there an Early Hearing Detection and Intervention Program in Saudi Arabia?	No	4	3.8
	Yes	86	81.1
	Unsure	16	15.1
How important do you think it is to screen all newborns for permanent hearing loss?	Somewhat important	4	3.8
	Very important	102	96.2
Hearing screening causes parents to feel excessive anxiety and/or concern.	No	54	50.9
	Yes	46	43.4
	Unsure	6	5.7
Are you aware that different health insurance plans cover audiological services, including hearing aids and cochlear implantation?	No	37	34.9
	Yes	33	31.1
	Unsure	36	34.0

Table 3 presents the answers related to hearing loss and hearing screening. The assessment of risk factors of permanent late-onset hearing loss in children showed that meningitis was the most mentioned factor (by 102 [96.2%] respondents), followed by family history of hearing loss (92; 86.8%), congenital syphilis (76; 71.7%), cytomegalovirus (CMV; 72 [67.9%]), and stay of ≥ 48 hours in the Neonatal Intensive Care Unit (NICU; 52 [49.1%]). Regarding the right age to conduct follow-up procedures for different scenarios, only 38 (35.8%) respondents correctly identified the right age (≤ 1 month old) for additional testing for a newborn who did not pass the hearing screening, 16 (15.1%) identified the right age (≤ 1 month old) in which a child can be definitely diagnosed with permanent hearing loss, 10 (9.4%) identified the right age (≤ 1 month old) in which a child can begin wearing hearing aids, and 66 (62.3%) identified the correct age group (from birth to 6 months of age) in which a child with permanent hearing loss be referred to early intervention services. Most participants (99; 93.4%) answered correctly regarding the right candidate for cochlear implants: infants with bilateral profound hearing loss. Only 44 (41.5%) participants provided correct answers regarding the best test (automated auditory brainstem response) for a hearing screening program.

**Table 3:** Knowledge related to hearing loss and screening

			N	%
**Risk factors for permanent late-onset hearing loss in children**		Meningitis*	102	96.2
		Congenital syphilis*	76	71.7
		Hypotonia	23	21.7
		Family history of hearing loss*	92	86.8
		Frequent colds	12	11.3
		≥ 48 hours in the Neonatal Intensice Care Unit (NICU)*	52	49.1
		Cleft palate*	25	23.6
		Cytomegalovirus (CMV)*	72	67.9
		Mother over 40 years of age	17	16.0
		Congenital heart disease	18	17.0
**Right age to conduct follow-up procedures**	A newborn not passing the hearing screening should undergo additional testing	≤ 1 month*	38	35.8
		1–3 months	54	50.9
		3–6 months	14	13.2
	A child can be definitely diagnosed with permanent hearing loss	≤ 1 month*	16	15.1
		1–3 months*	31	29.2
		3–6 months	33	31.1
		6–9 months	13	12.3
		9–12 months	13	12.3
	A child can begin wearing hearing aids	≤ 1 month*	10	9.4
		1–3 month*	18	17.0
		3–6 months	35	33.0
		6–9 months	27	25.5
		9–12 months	16	15.1
	A child with permanent hearing loss can be referred to early intervention services	≤ 1 month*	17	16.0
		1–3 month*	22	20.8
		3–6 months*	27	25.5
		6–9 months	24	22.6
		9–12 months	16	15.1
Opinions of the physicians about the right candidates for cochlear implants	Infants with bilateral mild-to-moderate hearing loss		18	17.0
	Infants with bilateral profound hearing loss*		99	93.4
	Infants with unilateral mild-to-moderate hearing loss		6	5.7
	Infants with unilateral profound hearing loss		53	50.0
Opinions of the physicians about the best tests for hearing screening programs	Automated auditory brainstem response (AABR)*		44	41.5
	Distortion product otoacoustic emission (DPOAE)		18	17.0
	Transient evoked otoacoustic emission (TEOAE)		40	37.7
	Tympanometry		4	3.8

**Note:** *Correct answer.

The participant's knowledge about hearing loss and hearing screening is shown in Table 4 . Only 19 (17.9%) respondents agreed or strongly agreed that children with recurrent conductive issues such as otitis media should receive hearing aids along with medication. A higher proportion of the participants (77; 72.6%) disagreed or strongly disagreed that children who receive hearing aids or cochlear implants could develop adequate speech and language skills without the need for speech therapy or auditory rehabilitation. Only 47 (44.4%) participants disagreed or strongly disagreed that children with a mild degree of SNHL do not need hearing aids. A total of 66 (62.3%) respondents disagreed or strongly disagreed that children with unilateral hearing loss may not be fitted with a hearing aid as they have one good (normal) ear. However, only 8 (7.6%) participants disagreed or strongly disagreed that the results of the auditory brainstem response (ABR) are preferred over behavioral testing (such as visual reinforcement audiometry, VRA) to assess hearing thresholds in children.

**Table 4:** Knowledge about hearing loss treatments

	Strongly disagree	Disagree	Unsure	Agree	Strongly agree
Children with recurrent conductive issues, such as otitis media, should receive hearing aids along with medication	37 (34.9%)	42 (39.6%)	8 (7.5%)	9 (8.5%)*	10 (9.4%)*
Children who receive hearing aids or cochlear implants can develop adequate speech and language skills without the need of speech therapy or auditory rehabilitation	51 (48.1%)*	26 (24.5%)*	6 5.7	9 (8.5%)	14 (13.2%)
Children with a mild degree of sensorineural hearing loss (SNHL) do not need hearing aids	20 (18.9%)*	27 (25.5%)*	20 (18.9%)	30 (28.3%)	9 (8.5%)
Children with unilateral hearing loss may not be fitted with hearing aids, as they have one good (normal) ear	29 (27.4%)*	37 (34.9%)*	14 (13.2%)	18 (17.0%)	8 (7.5%)
Auditory brainstem response (ABR) results are preferred over behavioral testing (such as visual reinforcement audiometry, VRA) to assess hearing thresholds in children	2 (1.9%)*	6 (5.7%)*	15 (14.2%)	29 (27.4%)	54 (50.9%)

**Note:** *Correct answer.

The total knowledge score was calculated by adding the score on each knowledge item, and correct answers were assigned a score of 1, while wrong answers were assigned a score of 0. The mean knowledge score was of 12.4 ± 2.12. The knowledge scores of the different types of ENT professionals were compared, and no statistically significant differences were observed ( *p* = 0.489). We also observed that female participants significantly presented higher knowledge scores compared with male participants ( *p* = 0.020). No statistically significant differences in knowledge scores were observed regarding participants with different years of experience ( *p* = 0.934), nor regarding those who worked in different places ( *p* = 0.300). However, we found that participants from the Eastern region presented significantly higher scores, and those from the Northern region, lower scores ( *p* = 0.021). The knowledge scores were significantly higher among those who examined > 50 children and between 11 and 50 children with SNHL compared to the scores of those who did not examine any children ( *p* = 00.8) ( Table 5 ).

**Table 5:** Comparison of knowledge scores regarding various sociodemographic characteristics

		N	Mean	Standard deviation	*p* -value
Type of ear, nose, and throat (ENT) professional	Consultant	35	12.57	±2.57	0.489
	Specialist	27	12.63	±1.78	
	Resident	43	12.02	±1.92	
	Teaching assistant	1	14.00		
Gender	Female	30	13.13	±1.83	0.020
	Male	76	12.08	±2.16	
Years of experience	≤ 1	15	12.60	±2.26	0.934
	2–5	42	12.33	±1.82	
	6–10	24	12.50	±2.21	
	> 11	25	12.20	±2.52	
Workplace	Medical school/university	19	12.21	±2.70	0.300
	Military medical services	27	12.89	±1.78	
	Ministry of Health	50	12.10	±2.00	
	Private pratice	4	11.50	±2.52	
	Other	6	13.50	±2.07	
Region of practice in Saudi Arabia	Central	44	12.34	±2.17	0.021
	Eastern	18	13.17	±1.92	
	Northern	3	9.00	±1.73	
	Southern	11	11.73	±1.95	
	Western	30	12.53	±1.98	
Number of children with permanent sensorineural hearing loss (SNHL) the participants examined in their practice last year	None	34	9.86	±1.46	0.008
	1–10	42	12.31	±2.03	
	11–50	23	12.71	±2.26	
	> 50	7	12.78	±1.78	

## Discussion

Hearing loss is a prevalent health issue with significant social and economic implications worldwide. In Saudi Arabia, where the population is rapidly growing and aging, understanding the knowledge and practice of ENT physicians regarding hearing screening and hearing loss management is crucial. In Saudi Arabia, ENT physicians undergo comprehensive medical training focusing on otolaryngology and audiology. They are trained to diagnose and treat various ear-related conditions, including hearing loss. However, the extent of their knowledge and expertise regarding hearing screening and hearing loss management may vary. The findings of the current study showed that most ENT physicians agreed that screening all newborns for permanent hearing loss is very important. It is expected that ENT physicians possess a solid understanding of the different types and causes of hearing loss, as well as of the available diagnostic tools and treatment options. 12 This includes knowledge of audiological assessments, such as pure-tone audiometry, tympanometry, and otoacoustic emissions testing, which are essential for an accurate diagnosis and appropriate intervention. 13 Additionally, knowledge of hearing aids, cochlear implants, and other assistive listening devices is crucial for effective hearing loss management. 14 The practice of ENT physicians in Saudi Arabia regarding hearing screening and hearing loss management is influenced by various factors, including healthcare policies, resources, and cultural considerations. 15 16 While many ENT physicians diligently apply evidence-based practices, some challenges may hinder optimal care delivery. 17

The findings of the current study show inadequate knowledge of hearing loss and its management. In a similar study conducted in Jordan, Zaitoun et al. 11 reported high awareness regarding hearing screening programs, but limited knowledge regarding hearing loss management and testing. Our findings revealed a wide variation in the respondents' knowledge regarding the risk factors for late onset hearing loss in children. Our respondents identified CMV, meningitis, mothers older than 40 years, and family history as the 4 most important risk factors. This was consistent with previous research, 11 18 which found that meningitis was the most recognized factor, followed by a family history of hearing loss and a history of CMV. Congenital CMV is a leading cause of non-genetic hearing loss in children, which may occur when a pregnant woman is infected with CMV for the first time during pregnancy or has a reactivation of a previous CMV infection, and then passes the virus to her developing fetus. 19 20

The findings of the present study show that physicians who examined a higher number of children had significantly higher knowledge. This could be because these physicians have had more opportunities to observe, diagnose, and manage cases related to hearing impairment. Through this exposure, they develop a deeper understanding of the condition, its causes, and its risk factors. Experienced physicians are often better at accurately diagnosing the underlying causes of hearing loss. 15 They may be more skilled at conducting thorough assessments, ordering appropriate diagnostic tests, and interpreting the results. This precision in diagnosis is crucial to identify risk factors and tailor treatment or management plans. Physicians, who are primarily responsible for children's hearing loss management, should be well-versed in such risk factors and practice constant vigilance in screening, monitoring, and referrals. The availability of comprehensive hearing screening programs for all age groups may be limited, especially in remote areas of Saudi Arabia. 21 Lack of universal hearing screening can result in delayed detection of hearing loss and subsequent intervention, leading to potential adverse effects on the individuals' communication skills and quality of life. 22 23 24 There was a lack of understanding among ENT doctors about the optimal age for audiological testing and therapy. When asked when they thought it was appropriate to conduct follow-up testing on infants who had failed the newborn hearing screening, most participants said after 1 month of age. However, 66 (62.3%) participants who were asked about the age range in which they would recommend beginning early intervention therapy for a child with permanent hearing loss answered correctly. Most participants knew about the right candidates for cochlear implants. According to the American Academy of Audiology's guidelines for pediatric amplification, 25 even mild hearing loss in children warrants fitting hearing aids to guarantee effective language and speech acquisition. 26 27 28 This contrasts with the more stringent criteria for hearing aid fitting for adults. 29 Loss of sound localization is one of the negative effects of unilateral hearing loss on a child's linguistic development. 30 Therefore, it is recommended that children with unilateral or mild hearing loss use hearing aids.

Conductive hearing loss is commonly misunderstood and handled as a transient ailment, which is partly true, but it is more problematic in the case of children. There is a window of opportunity for a child's language and speech development, and problems such as chronic otitis media can interfere with that growth and postpone it. 24 To guarantee healthy language and speech development, it is advised that children with recurrent conductive issues use hearing aids. 31 In Saudi Arabia, ENT physicians should actively engage in raising awareness about the importance of regular hearing screenings and early intervention. Educating patients and their families about the impact of untreated hearing loss can help ensure timely referrals and appropriate management. 32 Staying updated with the latest advancements in audiological technology, including hearing aids and cochlear implants, enables ENT physicians to provide the most suitable treatment options tailored to the individual's needs. Familiarity with the benefits and limitations of these devices ensures optimal patient outcomes. Promoting collaboration among ENT physicians, audiologists, and other relevant healthcare professionals through forums, meetings, and research initiatives can facilitate knowledge sharing and improve patient care outcomes. 33 Encouraging the development and implementation of national guidelines and policies related to hearing screening and hearing loss management can standardize practices and ensure consistent, evidence-based care across the country. 34 Launching targeted campaigns to raise public awareness about the importance of regular hearing screening sessions, early intervention, and the available treatment options can empower individuals and encourage them to seek medical help in a timely manner. 10

The limitations of the present study include its reliance on self-reported data, which could not be independently verified. Since taking part in the survey was entirely optional, there is a chance of selection bias. The participants may be physicians with a vested interest in or knowledge of the topic at hand, leading to an inflated picture of the state of ENT knowledge and practice. Two other issues with self-reported information are recall bias and social desirability bias. It is possible that doctors will fudge their answers or recall information in a way that makes them look better. In addition, closed-ended questions are frequently used in questionnaire surveys, which can hinder in-depth comprehension. To capture the complete spectrum of knowledge and practices related to complex topics such as hearing loss and interventional programs, it is necessary to provide in-depth explanations and open-ended responses. So, while questionnaire surveys might provide some useful information, it is necessary to supplement them with other research methods, such as interviews or observational studies, to provide a fuller picture of ENT physicians' knowledge and practices in this field.

## Conclusion

One of the main factors that lead to increased hearing loss consequences and progression is inadequate knowledge and practice of hearing screening and hearing loss management modalities, and the current study showed excellent knowledge about the significance of hearing screening. However, it also showed that there is an ongoing debate in many aspects of hearing screening. Moreover, it has been shown that the present study was limited in the assessment of these aspects especially among ENT physicians in Saudi Arabia. More research should be done to assess the practice of hearing screening in Saudi Arabia. Our recommendation is the provision of more educational sessions about the recent guidelines on hearing loss screening.

## References

[1] Cruickshanks K J Tweed T S Wiley T L Klein B EK Klein R Chappell R The 5-year incidence and progression of hearing loss: the epidemiology of hearing loss study Arch Otolaryngol Head Neck Surg 2003 129 10 1041 1046 10.1001/archotol.129.10.1041 14568784

[2] Mehl A L Thomson V The Colorado newborn hearing screening project, 1992-1999: on the threshold of effective population-based universal newborn hearing screening Pediatrics 2002 109 01 E7 10.1542/peds.109.1.e7 11773575

[3] Fortnum H M Summerfield A Q Marshall D H Davis A C Bamford J M Prevalence of permanent childhood hearing impairment in the United Kingdom and implications for universal neonatal hearing screening: questionnaire based ascertainment study BMJ 2001 323 7312 536 540 10.1136/bmj.323.7312.536 11546698 PMC48157

[4] World Health Organization Deafness and hearing lossAvailable from:https://www.who.int/health-topics/hearing-loss#tab=tab_1

[5] World Health Organization Deafness and hearing loss. Key FactsAvailable from:https://www.who.int/news-room/fact-sheets/detail/deafness-and-hearing-loss

[6] Wrobel C Zafeiriou M P Moser T Understanding and treating paediatric hearing impairment EBioMedicine 2021 63 103171 10.1016/j.ebiom.2020.103171 33422987 PMC7808910

[7] Davis A Bamford J Wilson I Ramkalawan T Forshaw M Wright S A critical review of the role of neonatal hearing screening in the detection of congenital hearing impairment Health Technol Assess 1997 1 10 i iv, 1–176 9483157

[8] Bafaqeeh S A Zakzouk S M al Muhaimeid H Essa A Relevant demographic factors and hearing impairment in Saudi children: epidemiological study J Laryngol Otol 1994 108 04 294 298 10.1017/s0022215100126581 8182312

[9] Daghistani K J Jamal T S Zakzouk S M The management of hearing impaired Saudi children: An epidemiological survey Bahrain Med Bull 2002 24 01 7 12Available from:https://www.bahrainmedicalbulletin.com/march_2002/management.pdf

[10] Alshawi Y A Al-Gazlan N Alrawaf F Almuhawas F Value of Newborn Hearing Screening on Early Intervention in the Saudi Population and Review of International Records Cureus 2019 11 10 e5990 10.7759/cureus.5990 31807378 PMC6876919

[11] Zaitoun M Rawashdeh M AlQudah S ALMohammad H Nuseir A Al-Tamimi F Knowledge and Practice of Hearing Screening and Hearing Loss Management among Ear, Nose, and Throat Physicians in Jordan Int Arch Otorhinolaryngol 2021 25 01 e98 e107 10.1055/s-0040-1709112 33542759 PMC7851363

[12] American Academy of Pediatrics, Joint Committee on Infant Hearing Year 2007 position statement: Principles and guidelines for early hearing detection and intervention programs Pediatrics 2007 120 04 898 921 10.1542/peds.2007-2333 17908777

[13] López-Vázquez M Berruecos P Lopez L E Cacho J Attitude and knowledge of hearing loss among medical doctors selected to initiate a residency in Mexico Int J Audiol 2009 48 03 101 107 10.1080/14992020802355882 19283581

[14] Holt R F Assistive Hearing Technology for Deaf and Hard-of-Hearing Spoken Language Learners Educ Sci (Basel) 2019 9 02 153 10.3390/educsci9020153 34113551 PMC8189502

[15] Alqudah O Alqudah S Al-Bashaireh A M Alharbi N Alqudah A M Knowledge, attitude and management of hearing screening in children among family physicians in the Kingdom of Saudi Arabia PLoS One 2021 16 08 e0256647 10.1371/journal.pone.0256647 34464417 PMC8407574

[16] Almatrafi M A Alsahaf N Kabli A Maksood L Alharbi K Alsharif A Predictors of Parental Recall of Newborn Hearing Screening Program in Saudi Arabia Healthcare (Basel) 2023 11 09 1357 10.3390/healthcare11091357 37174899 PMC10177918

[17] Yavari N Asghari F Shahvari Z Nedjat S Larijani B Obstacles of professional behavior among medical trainees: A qualitative study from Iran (2018) J Educ Health Promot 2019 8 193 10.4103/jehp.jehp_272_19 31807585 PMC6852278

[18] Ravi R Gunjawate D R Yerraguntla K Lewis L E Rajashekhar B A national survey of knowledge, attitude and practices among pediatricians towards newborn hearing screening in India Int J Pediatr Otorhinolaryngol 2017 95 9 14 10.1016/j.ijporl.2017.01.032 28576542

[19] Konopka W Śmiechura-Gańczarczyk M Pepaś R Cytomegalovirus infections in pregnant women as a risk of congenital deafness in a child Przegl Menopauz 2021 20 03 122 126 10.5114/pm.2021.109391 PMC8525258 34703412

[20] Boppana S B Pass R F Britt W J Stagno S Alford C A Symptomatic congenital cytomegalovirus infection: neonatal morbidity and mortality Pediatr Infect Dis J 1992 11 02 93 99 10.1097/00006454-199202000-00007 1311066

[21] Gosadi I M National screening programs in Saudi Arabia: Overview, outcomes, and effectiveness J Infect Public Health 2019 12 05 608 614 10.1016/j.jiph.2019.06.001 31248815

[22] Galhotra A Sahu P Challenges and Solutions in Implementing Hearing Screening Program in India Indian J Community Med 2019 44 04 299 302 10.4103/ijcm.IJCM_73_19 31802788 PMC6881890

[23] Patel H Feldman M Universal newborn hearing screening Paediatr Child Health 2011 16 05 301 310 10.1093/pch/16.5.301 22547950 PMC3114997

[24] American Academy of Pediatrics; American College of Obstetricians and Gynecologists Guidelines for Perinatal Care 5th ed. Elk Grove Village, IL/Washington, DC American Academy of Pediatrics/American College of Obstetricians and Gynecologists 2002

[25] Wall T C Senicz E Evans H H Woolley A Hardin J M Hearing screening practices among a national sample of primary care pediatricians Clin Pediatr (Phila) 2006 45 06 559 566 10.1177/0009922806290611 16893862

[26] Sabo D L The audiologic assessment of the young pediatric patient: the clinic Trends Amplif 1999 4 02 51 60 10.1177/108471389900400205 25425888 PMC4172161

[27] Hashemi M Akbari M E Razavi S S Saadat-Niaki A Hoseini Khameneh S M Evaluating resident physicians' knowledge, attitude, and practice regarding the pain control in cancer patients Iran J Cancer Prev 2015 8 01 1 10 25821565 PMC4360345

[28] Arnold C L Davis T C Humiston S G Bocchini J A Jr Bass P F III Bocchini A Infant hearing screening: stakeholder recommendations for parent-centered communication Pediatrics 2006 117(5 Pt 2):S341 S354 10.1542/peds.2005-2633N 16735261

[29] Vila P M Lieu J E Asymmetric and unilateral hearing loss in children Cell Tissue Res 2015 361 01 271 278 10.1007/s00441-015-2208-6 26004144 PMC4490007

[30] Lieu J EC Permanent unilateral hearing loss (UHL) and childhood development Curr Otorhinolaryngol Rep 2018 6 01 74 81 10.1007/s40136-018-0185-5 29651362 PMC5884900

[31] Erturk B B Genc G A Ozkan S Comparison of Hearing Screening Protocols for Universal Newborn Hearing Screening In Turkey J Int Adv Otol 2010 6 223 230

[32] Bennett R J Fletcher S Conway N Barr C The role of the general practitioner in managing age-related hearing loss: perspectives of general practitioners, patients and practice staff BMC Fam Pract 2020 21 01 87 10.1186/s12875-020-01157-2 32410580 PMC7226944

[33] Geese F Schmitt K-U Interprofessional Collaboration in Complex Patient Care Transition: A Qualitative Multi-Perspective Analysis Healthcare (Basel) 2023 11 03 359 10.3390/healthcare11030359 36766934 PMC9914692

[34] Alam S Satterfield A Mason C A Deng X Progress in Standardization of Reporting and Analysis of Data from Early Hearing Detection and Intervention (EHDI) Programs J Early Hear Detect Interv 2016 1 02 2 7 27840852 PMC5102258

